# Prognostic and therapeutic impact of RPN2-mediated tumor malignancy in non-small-cell lung cancer

**DOI:** 10.18632/oncotarget.2793

**Published:** 2015-01-20

**Authors:** Yu Fujita, Shigehiro Yagishita, Fumitaka Takeshita, Yusuke Yamamoto, Kazuyoshi Kuwano, Takahiro Ochiya

**Affiliations:** ^1^ Division of Molecular and Cellular Medicine, National Cancer Center Research Institute, Tsukiji, Chuo-ku, Tokyo 104-0045, Japan; ^2^ Division of Respiratory Diseases, Department of Internal Medicine, Jikei University School of Medicine, Nishi-shinbashi, Minato-ku, Tokyo 105-8471, Japan; ^3^ Depertment of Thoracic Oncology, National Cancer Center Hospital, Tsukiji, Chuo-ku, Tokyo 104-0045, Japan

**Keywords:** RPN2, NSCLC, RNAi, siRNA, malignancy

## Abstract

RNA interference (RNAi) is a powerful gene-silencing platform for cancer treatment. Previously, we demonstrated that ribophorin II (RPN2), which is part of the *N*-oligosaccharyl transferase complex, regulates docetaxel sensitivity and tumor lethal phenotypes in breast cancer. However, the molecular functions and clinical relevance of RPN2 in non-small-cell lung cancer (NSCLC) remain unknown. Here, we examined RPN2 expression in tumor specimens from recurrent NSCLC patients after resection (*n* = 32 and = 177) and assessed the correlation between RPN2 expression and various clinical features. We also investigated whether RPN2 affects cancer malignancy *in vitro* and tumor growth and drug resistance *in vivo*.

Our data show that RPN2 expression confers early and distant recurrence as well as poor survival in NSCLC patients. Furthermore, RPN2 silencing suppressed cell proliferation and invasiveness, and increased the sensitivity to chemotherapeutic drugs *in vitro*. Remarkably, we found that intrinsic apoptosis signaling is the mechanism of cell death involved with RPN2 knockdown. Strikingly, RPN2 silencing repressed tumorigenicity and sensitized the tumors to cisplatin treatment, which led to the longer survival of NSCLC-bearing mice.

In conclusion, these data suggest that RPN2 is involved in the regulation of lethal cancer phenotypes and represents a promising new target for RNAi-based medicine against NSCLC.

## INTRODUCTION

Lung cancer remains the most common cause of cancer-related death in the world. Approximately 85% of all lung cancers are categorized as non-small cell lung cancer (NSCLC). Many therapeutic strategies, including surgery, radiation therapy, and cytotoxic chemotherapy, are commonly used to treat lung cancer, either alone or in combination. Only a small percentage of newly diagnosed patients present with early stage NSCLC, and surgery remains the best therapeutic strategy for these patients. Approximately 70% of all newly diagnosed patients present with locally advanced or metastatic disease and require systemic chemotherapy [1]. With evidence that molecular targeted therapy is a major improvement over conventional chemotherapy when applied to appropriately selected patients, the evaluation for epidermal growth factor receptor (*EGFR*) mutations and anaplastic lymphoma kinase (*ALK*) gene rearrangements are considered to be the standard of care in advanced lung adenocarcinomas [2]. Despite the development of these novel targeted therapies, the median survival rate of individuals with advanced lung cancer remains poor because of drug resistance and tumor recurrence [3]. Therefore, understanding the critical molecular mechanisms underlying the development of these malignant phenotypes in NSCLC is important for developing novel and effective therapeutic strategies.

RNA interference (RNAi) is a natural endogenous mechanism for silencing gene expression that has recently become the focus of considerable attention because of its potential use in new drugs [4]. Cancer is a major target of RNAi-based therapy because oncogenes, mutated tumor suppressor genes, and several other genes that contribute to tumor progression and drug resistance are potentially important targets for gene silencing by small interfering RNAs (siRNAs) or short hairpin RNAs (shRNAs). Dozens of RNAi-based therapeutics are currently undergoing preclinical and clinical trials, and very few recent discoveries have attracted as much attention as the application of RNAi technology [5].

Previously, Honma *et al*. showed that ribophorin II (RPN2), which is part of an N-oligosaccharyl transferase complex, regulates the glycosylation of multi-drug resistance (MDR1, as well as ABCB1) [6]. RPN2 siRNA decreased the membrane localization of P-glycoprotein by reducing its glycosylation status and restored the sensitivity to docetaxel (DTX). Moreover, Takahashi *et al*. showed that RPN2 is multifunctional and tightly regulates tumor initiation and metastasis through the stabilization of mutant p53 in breast cancer cells [7]. Kurashige *et al*. showed that RPN2 expression is also able to predict the DTX response of esophageal squamous cell carcinoma [8]. Additionally, a recent study by Zhu *et al*. showed that RPN2 is highly expressed in the CD24^+^CD44^+^ cancer stem-like cells of pancreatic cancer [9]. However, it remains unclear whether these malignant phenotypes would be effective in other solid tumors, including lung cancer. Recently, we reported that a novel RNAi therapeutic platform against RPN2 demonstrated an efficient inhibition of lung tumor growth [10], but we have not yet revealed the molecular functions and clinical relevance of RPN2 in lung cancer. In this study, we examined RPN2 expression in tumor specimens from NSCLC patients who experienced recurrence after curative resection and assessed the correlation between RPN2 expression and the clinical features such as recurrence phenotypes and overall survival. Additionally, we also investigated whether RPN2 affected cancer malignancy *in vitro* as well as tumor growth and drug resistance *in vivo*. Our findings suggest that RPN2 may be a novel target for RNAi-based technology against NSCLC in clinical translational medicine.

## RESULTS

### High RPN2 expression in post-operative NSCLC patients is significantly associated with early and distant recurrence

**Figure 1:**
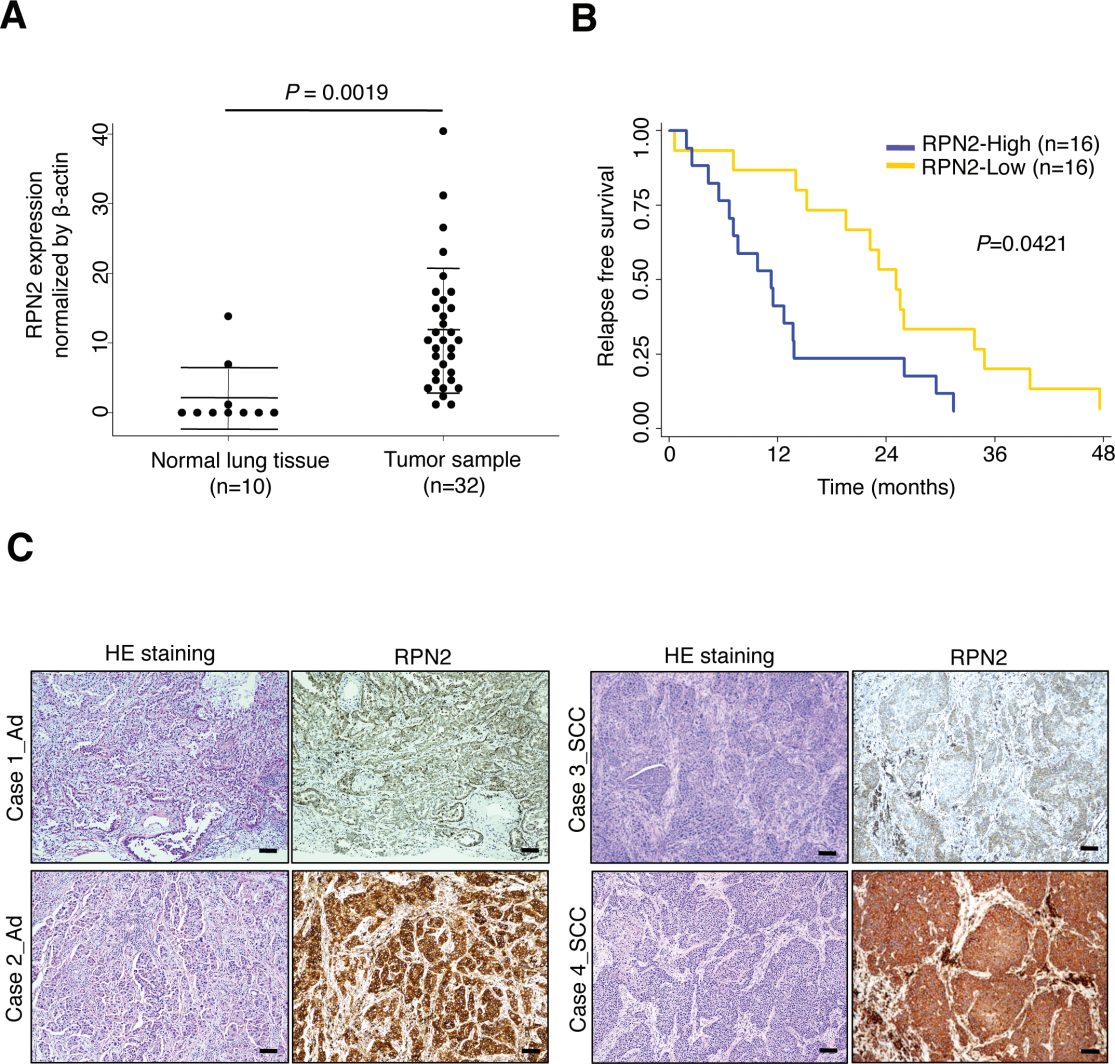
RPN2 confers early tumor relapse in NSCLC **(A)** qRT-PCR analysis of the expression levels of RPN2 in tumor samples (*n* = 32) and normal lung tissues (*n* = 10). **(B)** The relapse-free survival Kaplan-Meier curves were based on RPN2 expression, and the median RPN2 expression was used to define low and high expressers (*P* = 0.0421). **(C)** HE staining and immunohistochemical staining for RPN2 protein in representative tumors of lung cancer patients (adenocarcinoma (Ad) and squamous cell carcinoma (SCC)) are shown (scale bar 100 μm).

To explore the role of RPN2 in NSCLC, we analyzed the mRNA expression level of RPN2 using quantitative real-time PCR (qRT-PCR) in a cohort study of primary NSCLC tissues (32 cases) and adjacent normal lung tissues (10 cases) from patients with post-operative recurrence (Table 1). We observed an increase in RPN2 expression in the tumor tissue compared with normal tissues (Figure 1A; *P* = 0.0019). To further understand the potential biological significance of a high RPN2 expression level during lung cancer progression, we evaluated the correlation between the RPN2 expression profile and relapse-free survival in the cohort study. The Kaplan-Meier analysis indicated that high expression levels of RPN2 were significantly associated with poor relapse-free survival using the median value as the cut-off (Figure 1B; *P* = 0.0421). This statistical analysis revealed that there is a significant correlation between high RPN2 expression and distant recurrent metastasis (Table 1; *P* = 0.0339). We found no significant correlation between RPN2 expression and other clinicopathological features (Table 1). The immunohistochemical analysis revealed that RPN2 protein was weakly to strongly expressed in all of these specimens and localized in the cytoplasm. Additionally, there are no significant differences in the expression of RPN2 between adenocarcinoma and squamous cell carcinoma (Figure 1C). These findings suggest that a high RPN2 expression level in NSCLC is correlated with early recurrence and distant metastasis and may regulate lung cancer progression.

**Table 1:** Characteristics of the participating patients of a cohort study (*n* = 32)

Features	Total (*n* = 32)	RPN2		*P*-value
		Low (*n* = 16)	High (*n* = 16)	
Median Age (Range)	65.5 (44–75)	60.5 (46–75)	67.5 (44–74)	0.269
Gender				1
Male / Female	28 / 4	14 / 2	14 / 2	
Histological subtype				0.068
Adenocarcinoma	21	13	8	
Squamous cell carcinoma	11	3	8	
Smoking status				0.500
Never / Former or Current	7 / 25	4 / 12	3 / 13	
Pathological Stage (%)				0.379
Stage I	8	5	3	
Stage II	7	1	6	
Stage III	17	10	7	
Stage IV	0	0	0	
RPN2 mRNA expression				0.000
High	16	0	16	
Low	16	16	0	
Recurrence				0.038
Local (lung, local lymph node)	18	11	5	
Distant (other organs)	14	5	11	

### RPN2 regulates various malignant phenotypes of NSCLC *in vitro*

**Figure 2:**
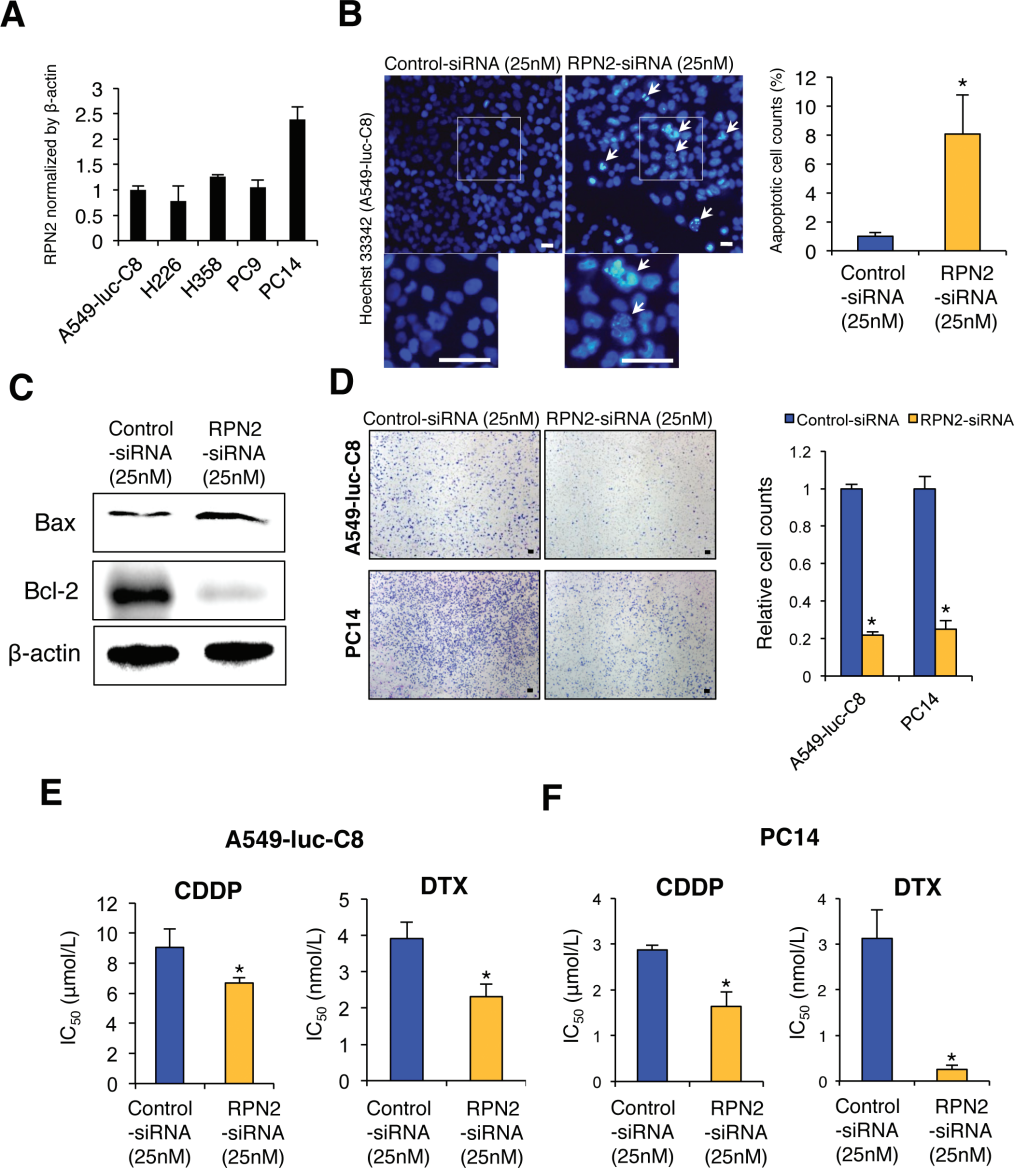
RPN2 regulates malignant phenotypes of lung cancer cells *in vitro* **(A)** qRT-PCR analysis of the expression levels of RPN2 in human lung cancer cell lines. **(B)** Representative images (left panels, pictures) and quantification (right panels, graphs) of Hoechst 33342-stained sections after each siRNA transfection (scale bar 50 μm). **(C)** A549-luc-C8 cells were transiently transfected with each siRNA. Bax and Bcl-2 expression was detected using immunoblot analysis. β-actin was used as a loading control. **(D)** Representative images (left panels, pictures) and quantification (right panels, graphs) of the effect on cell invasion in A549-luc-C8 or PC14 cells transfected with each siRNA (scale bar 100 μm). The invasive values were normalized to the values from control cells. **(E and F)** IC_50_ after CDDP or DTX treatment in A549-luc-C8 **(E)** or PC14 cells **(F)** transfected with each siRNA. *P < 0.05.

To investigate the functional effects of regulating RPN2 in NSCLC cells, we first verified the expression of RPN2 mRNA in various lung cancer cell lines (Figure 2A). We found a higher expression of RPN2 in PC14 cells than in A549-luc-C8, H226, H358, or PC9 cells. In this study, we selected two cell lines, A549-luc-C8 (mutant k-ras) and PC14 (mutant p53), in which to analyze the detailed functions of RPN2 in NSCLC. Then, we conducted siRNA-based silencing of RPN2 in these two cell lines. The reduced expression of RPN2 was verified by qRT-PCR (Supplementary Figure 1A) and western blot analysis (Supplementary Figure 1B). As shown in Supplementary Figure 1C, RPN2 siRNA inhibited the cell proliferation of A549-luc-C8 and PC14 cells. To evaluate the mode of cell death induced by RPN2 silencing in lung cancer cells, we performed assays for apoptosis using Hoechst staining and western blot analyses in these cells after RPN2 siRNA transfection. The number of apoptotic cells, as assessed by Hoechst 33342 staining, was significantly higher in these cell types after RPN2 siRNA transfection (Figure 2B & Supplementary Figure 2A). Additionally, we assessed the expression of Bax and Bcl-2, the two principal genes involved in intrinsic apoptosis control, by western blot analysis. The results showed that Bax expression increased and Bcl-2 expression decreased in the two cell lines after RPN2 siRNA transfection compared with negative control siRNA transfection (Figure 2C & Supplementary Figure 2B). These results suggest that the intrinsic apoptosis signaling pathway is the mode of cell death induced by RPN2 silencing in lung cancer cells. Next, we assessed the effects of RPN2 silencing on cell invasion and drug sensitivity. Remarkably, we observed that siRNA-based silencing of RPN2 in these cell lines resulted in a significant decrease in invasion and sensitized the response of the cells to Cisplatin (CDDP) and DTX (Figure 2D, 2E, 2F). Thus, the results of all of these experiments suggest that RPN2 silencing inhibits various malignant phenotypes of NSCLC *in vitro*.

### RPN2 silencing contributes to the inhibition of tumor growth and drug resistance *in vivo*

**Figure 3:**
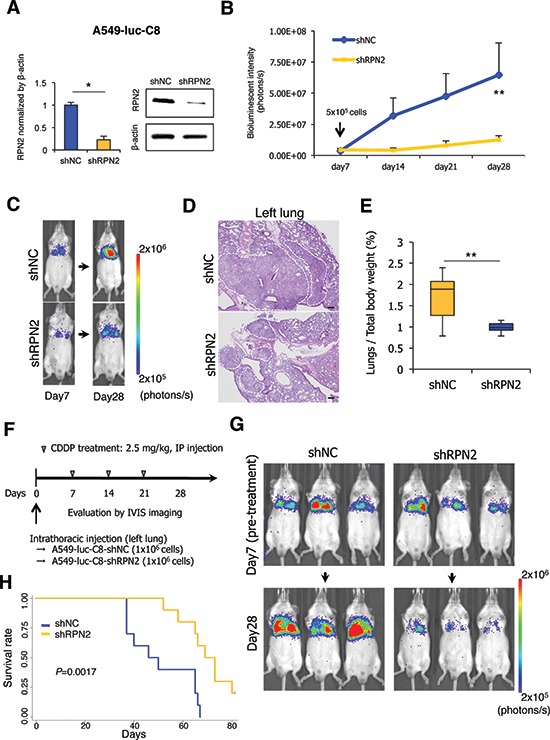
RPN2 regulates tumorigenicity and drug resistance *in vivo* **(A)** qRT-PCR and immunoblot analysis of the expression levels of RPN2 in A549-luc-C8-shNC and -shRPN2 cells. (B and C) The bioluminescent change emitted from the whole body of the mouse **(B)** and representative bioluminescence images of lung tumor growth in each group (10 mice per group) **(C)** are shown. **(D)** Representative pictures of HE staining of the left lung are shown (scale bar 200 μm). White dotted lines indicate the area containing the tumors. **(E)** The ratio of lung weight per total body weight in mice was calculated. **(F)** The protocol of the repeat intraperitoneal injection (IP injection) of CDDP (10 mice per group). **(G)** Representative bioluminescence images of lung tumor growth after repeat administration of CDDP are shown. **(H)** The overall survival rate of each group was estimated by the Kaplan-Meier method (*P* = 0.0421). *P < 0.05. **P < 0.01. NC, negative control.

To validate the function of RPN2 as a regulator of lung tumor progression *in vitro*, we first established stable clones of A549-luc-C8 cells expressing shRNA against RPN2 (A549-luc-C8-shRPN2) or control shRNA (A549-luc-C8-shNC). The reduced expression of RPN2 was verified by qRT-PCR and western blot analysis (Figure 3A). We examined the effects of A549-luc-C8-shRPN2 cells on tumorigenicity and drug resistance using lung cancer xenograft models established by direct intrathoracic injection (left lung) into scid mice. Using bioluminescence imaging, we found that the primary lung tumor growth of A549-luc-C8-shRPN2 cells (*n* = 10) was less than that of A549-luc-C8-shNC cells (*n* = 10) (Figure 3B, 3C). Histological analyses also revealed that RPN2 silencing represses the tumor growth of this lung cancer cell line (Figure 3D). Additionally, the weight of the lungs significantly decreased in the A549-luc-C8-shRPN2-bearing mice group (Figure 3E). However, none of the mice from either group developed distant metastases during the observation period; therefore, we could not investigate the metastatic phenotypes regulated by RPN2 *in vivo*. Next, to investigate whether RPN2 silencing affects the drug resistance of A549-luc-C8 cells to CDDP *in vivo*, tumors were initiated in scid mice and then followed by an intraperitoneal injection of 2.5 μg/kg CDDP once a week for three weeks (Figure 3F). At four weeks after orthotopic transplantation, the tumors formed in the A549-luc-C8-shRPN2-bearing mice (*n* = 10) were significantly smaller compared with the tumors in the A549-luc-C8-shNC-bearing mice (*n* = 10) based on bioluminescence imaging results (Figure 3G). All the mice were evaluated for survival, and A549-luc-C8-shRPN2-bearing mice survived longer than the A549-luc-C8-shNC-bearing mice (Figure 3H; *P* = 0.0149). Together, these results indicate that RPN2 silencing confers therapeutic and survival advantages to lung cancer cells *in vivo*.

### Survival outcomes of RPN2 expression in a validation cohort study

**Figure 4:**
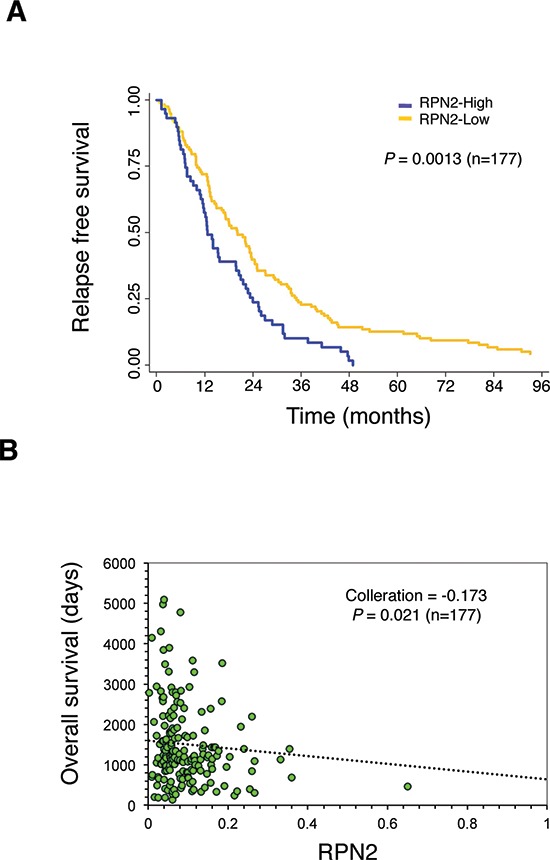
The clinical relevance of RPN2 in a validation cohort study of NSCLC **(A)** Relapse-free survival outcome correlated with RPN2 expression in a validation cohort study. (*P* = 0.0013, *n* = 177). **(B)** Scatter plots between RPN2 gene expression and overall survival (*P* = 0.021, *n* = 177).

To further understand the potential biological significance and clinical relevance of RPN2 expression in lung cancer progression, we evaluated the correlation between RPN2 expression and survival in tumor specimens from 177 NSCLC patients who experienced recurrence after curative resection (Supplementary Table 1). The RPN2 expression levels were measured by qRT-PCR, and the Kaplan-Meier analysis indicated that high expression levels of RPN2 were significantly correlated with poor relapse-free survival (Figure 4A; *P* = 0.0013). Furthermore, we examined the association between RPN2 and overall survival. Based on the mRNA expression levels and clinical data, we observed a significant negative correlation between RPN2 expression and overall survival (Figure 4B; *P* = 0.021). In conclusion, our data suggest that RPN2 is a novel potential therapeutic target for NSCLC.

## DISCUSSION

In the present study, we show that the RPN2 gene is significantly correlated with early and distant tumor recurrence as well as poor survival in NSCLC patients. Furthermore, RPN2 silencing by siRNAs suppresses cell proliferation and invasiveness and increases the sensitivity of lung cancer cells to chemotherapeutic drugs *in vitro*. Remarkably, we found that the intrinsic apoptosis signaling pathway is the mechanism of cell death induced by RPN2 silencing in lung cancer cells. Notably, RPN2 silencing by shRNAs repressed lung tumor growth and sensitized the lung cancer cells to CDDP treatment, which led to the longer survival of lung cancer-bearing mice. These data suggest that RPN2 promotes lung cancer cell malignancy and represents a promising novel target for RNAi-based therapeutics against NSCLC.

RPN2 is a key regulator in modulating DTX sensitivity in breast cancer cells through the glycosylation of P-glycoproteins [6]. Moreover, RPN2 silencing reduced the glycosylation of MDR1 and decreased its membrane localization, which thereby sensitized the cancer cells to DTX. Recently, it was also reported that RPN2 expression may be a potential therapeutic target for drug resistance and a promising prognostic biomarker for various types of cancer [8, 11]. We have also recently demonstrated that RPN2 regulates cancer stem cell properties through the functional suppression of glycogen synthase kinase 3β (GSK3β) in breast cancer cells [7]. Based on this strong evidence supporting these novel functions of RPN2, we suggest that RPN2 expression not only controls DTX sensitivity but also plays multifunctional roles in various types of cancer cells. In this study, we show that RNAi-mediated knockdown of RPN2 in NSCLC resulted in cell death *in vitro* and *in vivo* by downregulating the anti-apoptotic intrinsic pathway independent of k-ras or p53 status. It has been reported that the upregulation of anti-apoptotic Bcl-2 family genes is an important regulator of tumor malignancy and drug resistance [12–14]. In our study, RPN2 silencing by siRNA in lung cancer cells selectively regulated the expression of two principal genes involved in intrinsic apoptosis control. We speculate that RPN2 confers lung cancer cell growth and various lethal phenotypes by modulating anti-apoptotic intrinsic signaling.

The invasion-metastasis cascade is a biological sequence of steps that describes how cancer cells from a primary tumor can form distant metastases. In this study, we also show that RPN2 modulates the metastatic phenotypes in recurrent NSCLC patients as well as the cell invasion ability of lung cancer cells. Although the molecular mechanism underlying the cell invasiveness related to RPN2 remains unknown, our recent study reported that glycosylated CD63 by RPN2 plays an important role in breast cancer cell invasiveness by regulating MDR1 localization [15]. CD63 was the first tetraspanin to be identified and regulates the biological function of various solid tumors, especially those with metastatic potential [16]. Further studies are needed to elucidate whether the glycosylation status of CD63 plays a role in tumor cell invasion and metastatic phenotypes regulated by RPN2 in NSCLC.

Our preclinical studies demonstrate the clinical relevance of RPN2-mediated cancer malignancy in various types of solid cancer. Currently, we are conducting a clinical phase I study of siRNA targeting RPN2 in advanced breast cancer patients in Japan and anticipate that this study will generate novel information that will be useful for NSCLC treatment. It is important for successful clinical application to assess the safety and tolerability of RPN2-siRNA in human body. Thus, RNAi-based approaches are a promising therapeutic strategy for cancer treatment. The crucial problems impeding the development of RNAi-based therapeutics include effective delivery to target sites, therapeutic potency, and elimination of off-target effects [17–19]. In particular, investigators should pay much attention to the off-target effects induced by siRNA treatment. In this study, we confirmed that negative control siRNA could not affect the expression of RPN2 and cell survival and proliferation of A549-C8-luc and PC14 cells. Furthermore, RPN2-siRNA could down-regulate RPN2 expression with high specificity and inhibit the cell growth of these cells (Supplementary Figure 1). Therefore, potential of off-target events caused by RPN2-siRNA treatment could not be concerned in these experiments. Notably, we previously reported that a novel RNAi-based strategy using aerosol delivery successfully resulted in inhibition of lung tumor growth without any significant signs of toxicity *in vivo* [10]. Therefore, we believe that RNAi-based therapy will play a key role in the future treatment of lung cancer.

In conclusion, this study suggests that RPN2 silencing contributes to the regulation of lethal cancer phenotypes and provides a recurrence and survival advantage in NSCLC. RNAi-based approaches against the RPN2 gene may open new avenues for NSCLC treatment and improve the clinical outcome of the patients.

## MATERIALS AND METHODS

### Reagents

Mouse monoclonal anti-RPN2 (A-1)(sc-166421), mouse monoclonal anti-Bcl-2 (C-2)(sc-7382), and rabbit polyclonal anti-Bax (N-20)(sc-493) were purchased from Santa Cruz Biotechnology (Dallas, TX). Mouse monoclonal anti-actin (C4)(MAB1501) was purchased from Millipore (Billerica, MA). Hoechst 33342 dye was obtained from Dojindo (Kumamoto, Japan). The siRNA targeting human RPN2 mRNA (target sequences of 5′-GGCCACUGUUAAACUAGAACA-3′ and 5′-UUCUAGUUUAACAGUGGCCUG-3′) was purchased from Bonac (Kurume, Japan). The Allstars Negative Control siRNA (1027281) was purchased from Qiagen (Hilden, Germany). CDDP was obtained from Yakult Honsha Co., Ltd. (Tokyo, Japan). DTX was obtained from SANOFI K.K. (Tokyo, Japan).

### Cell culture and transient transfection

A549-luc-C8 cells were purchased from PerkinElmer (Norwalk, CT). H226, H358, PC9, and PC14 cell lines were purchased from the American Type Culture Collection (ATCC, Manassas, VA). These cells were cultured in RPMI 1640 medium with 10% heat-inactivated fetal bovine serum (FBS) and an antibiotic-antimycotic solution (Invitrogen, Carlsbad, CA) at 37°C in 5% CO_2_. The siRNA transfection at 25 nM was conducted with DharmaFECT 1 reagent (Thermo Scientific, San Jose, CA) according to the manufacturer's protocol. Lentiviral vector transfection (500 ng) was conducted with Lipofectamine LTX reagent (Invitrogen) according to the manufacturer's protocol.

### RNA extraction, reverse transcription, and quantitative real-time RT-PCR (qRT-PCR)

Total RNA was extracted from cultured cells or human lung tissues using QIAzol and the miRNeasy Mini Kit (Qiagen) according to the manufacturer's protocol. The purity and concentration of all RNA samples were quantified using a NanoDrop ND-1000 spectrophotometer (Thermo Scientific). Detection of RNA from human lungs was performed using the Agilent Bioanalyzer 2100 (Agilent Technologies, Santa Clara, CA). Prior to the analysis, total RNA was prepared using an Agilent RNA 6000 Pico kit (Agilent Technologies) according to the manufacturer's protocol. All clinical samples exhibited RNA Integrity Numbers > 6.0. The reverse transcription reaction was performed using a High-Capacity cDNA Reverse Transcription Kit (Applied Biosystems, Foster City, CA) and a random hexamer primer. The synthesized cDNAs were quantified by SYBR Green I qRT-PCR. Quantitative real-time reverse transcription-PCR (qRT-PCR) analysis was conducted using primers for human RPN2 (forward: 5′-CTTCCAGAGCCACTGTCCTC-3′; reverse: 5′-CCGGTTGTCACCTTCAACTT-3′). β-actin (forward: 5′-ATTGCCGACAGGATGCAGA-3′; reverse: 5′-GAGTACTTGCGCTCAGGAGGA-3′) was used for normalization. The relative amounts of RPN2 were measured using the 2(−Delta Delta C(T)) method. The reactions were performed using a ABI 7300 Real-Time PCR System (Applied Biosystems). All reactions were performed in triplicate.

### Immunoblot analysis

SDS-PAGE gels were calibrated using Precision Plus protein standards (161–0375) (Bio-Rad, Hercules, CA), and anti-RPN2 (1:100), anti-Bcl-2 (1:200), anti-Bax (1:100), and anti-actin (1:1,000) were used as the primary antibodies. The dilution ratio of each antibody is indicated in parentheses. Two secondary antibodies (peroxidase-labeled anti-mouse and anti-rabbit antibodies) were used at a dilution of 1:10,000. Bound antibody was visualized by chemiluminescence using an ECL Plus Western blotting detection system (GE Healthcare, Piscataway, NJ), and luminescent images were analyzed with a LuminoImager (LAS-3000; Fuji Film Inc., Tokyo, Japan).

### Cell viability assay

A Cell Counting Kit-8 (CCK-8) (Dojindo) was used in the cell viability assay. Three thousand cells per well were seeded into 96-well plates. The following day, the cells were replenished with fresh medium containing 25 nM of siRNA. The cells were used for cell proliferation assays or cytotoxicity assays. For the cell proliferation assay, 10 μl of CCK-8 solution was added to each well after 24 h, 48 h, 72 h, 96 h, and 120 h. The plate was further incubated for 2 h at 37°C. The absorbance at 450 nm was measured using an Envision Multilabel Reader (PerkinElmer). For cytotoxicity assays, the medium was aspirated off the following day and replaced with fresh media containing CDDP and DTX at different concentrations. After three days of culture, the plate was assayed by adding 10 μl of CCK-8 solution to each well, and the plate was further incubated for 2 h at 37°C. The absorbance at 450 nm was measured using an Envision Multilabel Reader. Cell viability is expressed as a percentage of the control (untreated) cell viability. For each concentration of CDDP and DTX, the mean values of the mean absorbance rates from eight wells were calculated. The IC_50_ (concentration of drug needed to inhibit cell growth by 50%) was generated from the dose-response curves for each cell line.

### Cell invasion assay

For the invasion assay, 1 × 105 cells were placed in the upper chamber of each insert in 24-well Biocoat Matrigel invasion chambers (8 μm pore size; BD Biosciences). For the assay, the cells were trypsinized and resuspended in RPMI 1640. Medium supplemented (750 μL) with 10% FBS was injected into the lower chambers. The cells were incubated at 37°C as follows: 24 h for A549-luc-C8 and PC14 cells in the invasion assays. Subsequently, any cells remaining in the top chambers or on the upper membrane of the inserts were carefully removed. The cells that migrated through the membrane and adhered to the lower surface of the membrane were fixed with methanol and stained with Diff Quick staining solution. For quantification, the cells were counted under a microscope in four random fields.

### Lentiviral shRNA transduction

Cell lines stably expressing RPN2 shRNA or control non-target shRNA were established using a vector-based shRNA technique and the Human RPN2 shRNA target 5′-GCCACTTTGAAGAACCCAATC-3′ and control shRNA target 5′-GAAATGTACTGCGCGTGGAGAC-3′. Briefly, each fragment was subcloned into pGreenPuro (System Biosciences, Mountain View, CA). Recombinant lentiviruses were produced following the manufacturer's instructions. In knockdown experiments, A549-luc-C8 cells were infected with recombinant lentiviruses expressing control shRNA (shNC) or shRNA against RPN2 (shRPN2). After transfection, cell lines were generated by selection with 4 μg/μl puromycin (Invitrogen).

### Animal studies

Animal experiments were performed in compliance with the guidelines of the Institute for Laboratory Animal Research, National Cancer Center Research Institute (Number: T12-004). Six- to seven-week-old male C.B-17/Icr-scid/scidJcl mice (CLEA, Tokyo, Japan) were used in the experiments. Intrathoracic injections were given following a previous protocol with minor modifications [20]. The cells were resuspended in PBS as a half volume of inoculum and diluted with an equal volume of growth factor-reduced Matrigel (356231; BD Biosciences). For *in vivo* imaging, the mice were administered 150 μg/kg D-luciferin (Promega, Madison, WI) by intraperitoneal injection. Ten minutes later, photons from the whole body of the animal were counted by measuring the bioluminescence with an IVIS imaging system (PerkinElmer) according to the manufacturer's instructions. The data were analyzed using the LIVINGIMAGE 4.2 software (PerkinElmer). Lung cancer development was monitored twice a week *in vivo* by bioluminescent imaging. In the repeated administration study, the treatment (2.5 μg/kg of CDDP) was performed on days 7, 14, and 21 (once a week for three weeks, three treatments total).

### Clinical lung tissue samples

The study protocol was approved by the Institutional Review Board at the National Cancer Center (Number: 2012-054). All materials were obtained with written informed consent and were provided by the National Cancer Center Biobank. All human lung tissue samples were from the resected lungs of lung cancer patients who had recurrence after pulmonary resection at the Department of Thoracic Surgery in the National Cancer Center Hospital between 1997 and 2010. We selected 32 lung tumor samples and 10 corresponding normal lung tissues for the cohort analysis, and 177 lung tumor samples were selected for the validated cohort study (Table 1 and Supplementary Table 1). In this study, patients who were diagnosed with NSCLC (adenocarcinoma or squamous cell carcinoma) were enrolled. Upon removal of the surgical specimen, the research personnel immediately transported the tissues to the Biobank. Tissues were stored at −80°C after snap freezing in liquid nitrogen. All tumors were reviewed by pathologists and were pathologically diagnosed according to the World Health Organization Classification of Tumors.

### Lung histological findings

Human and mouse lung tissues were fixed in 10% neutral buffered formalin, paraffin-processed, and sectioned at 5 μm. Formalin-fixed and paraffin-embedded slides were stained with hematoxylin and eosin (HE) or subjected to immunohistochemical (IHC) staining. For the IHC staining, antigen retrieval was performed by autoclave heating in a 10 mmol/l sodium citrate buffer (pH 6.0), and the endogenous peroxidase activity was blocked with the Immuno Pure Peroxidase Suppressor (Pierce, Chester, UK). The slides were incubated with RPN2 primary antibody at 4°C overnight. The next day, after washing, the samples were incubated with mouse peroxidase-conjugated anti-mouse IgG (ImmPRESS Reagent; Vector Labs, Burlingame, CA) for 1 h. The immunoreactions were visualized with diaminobenzidine.

### Statistical analyses

All experiments were repeated at least three times, and the results are expressed as the mean ± SD. The statistical analyses were mainly conducted using Student's *t*-test or Fisher's exact test. Pearson's correlation was estimated between RPN2 and overall survival. The Kaplan-Meier method was used to estimate survival as a function of time, and survival differences were analyzed by the log-rank test. All of the analyses were performed using PASW statistics version 18.0 (IBM Inc., Armonk, NY) and STATA version 12.0 software (Stata Corp, College Station, TX). Probability values < 0.05 indicated a statistically significant difference.

## SUPPLEMENTARY MATERIALS


