# Supplementary material for: Prognostic and therapeutic impact of RPN2-mediated tumor malignancy in non-small-cell lung cancer
Source: Oncotarget. 2015 Jan 20;6(5):3335–45. doi: 10.18632/oncotarget.2793 (PMC4413657; doi:10.18632/oncotarget.2793)
Supplement: Supplementary file 1 [file oncotarget-06-3335-s001.pdf]

SUPPLEMENTARY FIGURES AND TABLE

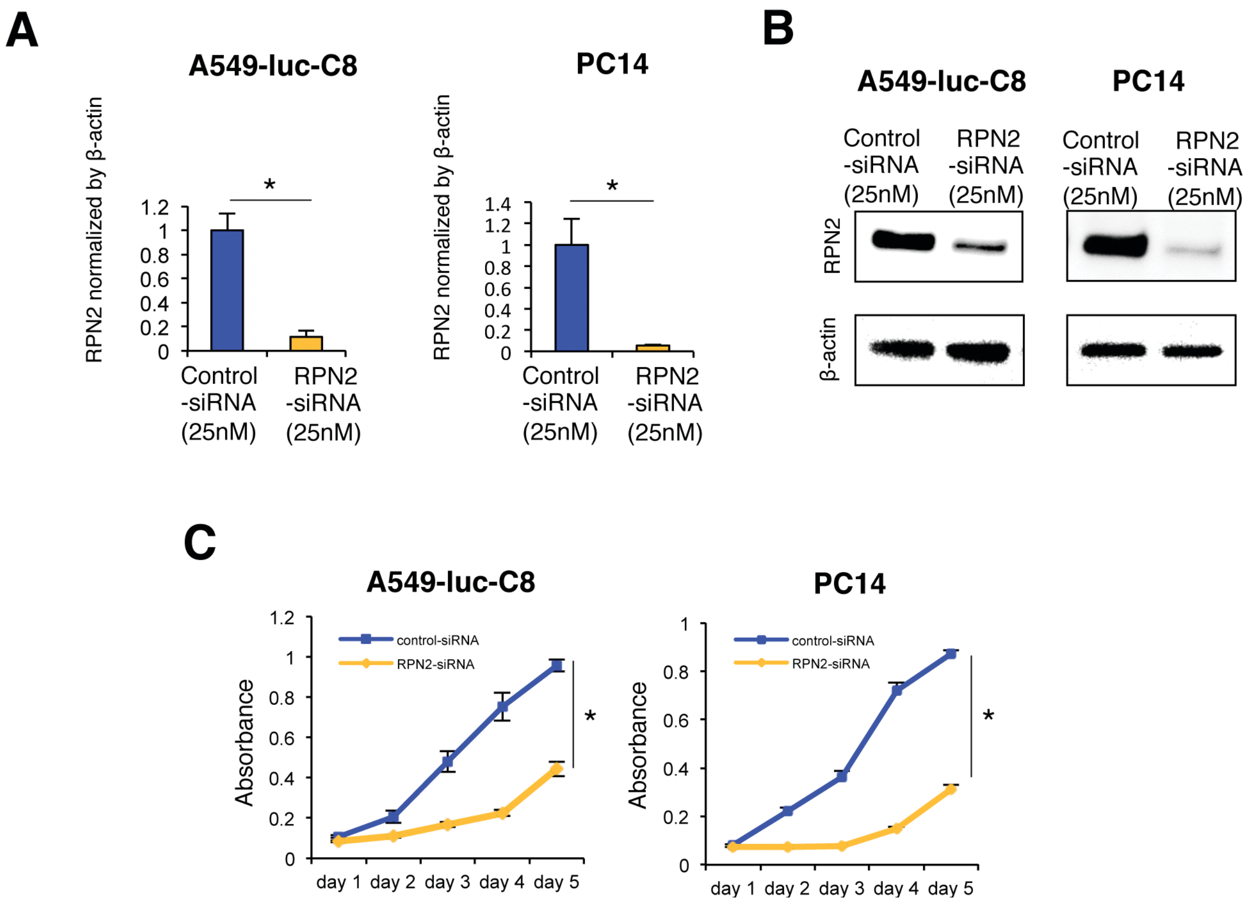

Supplementary Figure 1: RPN2 regulates cell proliferation of A549-luc-C8 and PC14 cells *in vitro.* (A and B) The reduced expression of RPN2 was confirmed by qRT-PCR (A), as well as by western blot analysis (B) in the two cell lines. **(C)** Cell proliferation assay in A549-luc-C8 or PC14 cells transfected with control or RPN2 siRNAs. *$P < 0.05$.

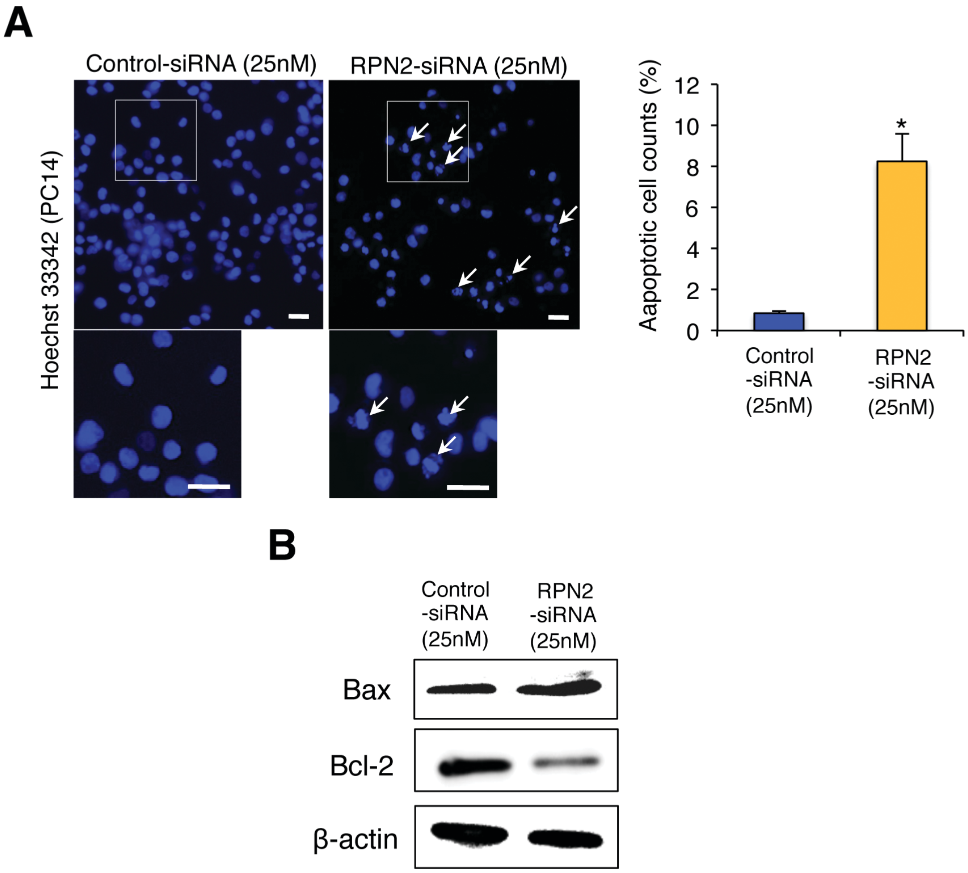

Supplementary Figure 2: An apoptosis assay using Hoechst staining and western blot analysis in PC14 cells after RPN2 siRNA transfection. **(A)** Representative images (left panels, pictures) and quantification (right panels, graphs) of Hoechst 33342-stained sections after each siRNA transfection (scale bar 50 μm). **(B)** PC14 cells were transiently transfected with each siRNA. Bax and Bcl-2 expression was detected using immunoblotting. β-actin was used as a loading control. $*P < 0.05$.

Supplementary Table 1: Characteristics of participating patients of a validation cohort study (*n* = 177)

Patient and Disease Characteristics	
**Number of patients**	**Validation cohort *n* = 177**
Median Age (Range)	65 (34-82)
Gender	
Male / Female	111 / 66
Histological subtype	
Adenocarcinoma	151
Squamous cell carcinoma	26
Smoking status	
Never / Former or Current	67 / 110
Pathological Stage (%)	
Stage I	57
Stage II	23
Stage III	97
Stage IV	0
